# Editorial: IoT, UAV, BCI empowered deep learning models in precision agriculture

**DOI:** 10.3389/fpls.2024.1399753

**Published:** 2024-05-01

**Authors:** Jian Lian, José Dias Pereira

**Affiliations:** ^1^ School of Intelligence Engineering, Shandong Management University, Jinan, China; ^2^ Instituto Politécnico de Setúbal, Escola Superior de Tecnologia de Setúbal, Setúbal, Portugal

**Keywords:** Internet of the Things, Unmanned Aerial Vehicle, deep learning, machine vision applications, precision agriculture, crop monitoring, plant disease recognition and classification

## Introduction

This Research Topic focuses the recent development in the Internet of Things and deep learning algorithms, including convolutional neural networks, transformer, and diffusion models, for precision agriculture in field and specialty crops. The 15 accepted papers include original research and review articles focusing novel deep learning algorithms, architectures, and applications of various instruments combined with the Internet of Things (IoT) and others advanced devices.

## Research Topic coverage

We collected two reviews and thirteen research papers on the Research Topic focused by this Research Topic. The authors of the accepted publications presented articles that cover mainly of the following topics: deep learning models for precision agriculture; deep learning, BCI, and UAV-based crop monitoring; plant disease recognition and classification; UAV and deep learning for plant species detection and classification; deep learning and the BCI-empowered UAV applications for precision agriculture and optimization for deep learning algorithms in Precision Agriculture. There was a total of 37 submitted papers, 15 were accepted and 22 were rejected, that means an acceptance rate around 40%. This editorial discusses AI advancements in categorization, segmentation, detection, monitoring, and route planning that are influencing agriculture globally.

## Image classification

Leaf disease classification needs improvement for precision agriculture applications. Han and Guo propose a new method for diagnosing leaf diseases in ligneous plants using an enhanced vision transformer model. The suggested method uses a multi-head attention module to record pictures and class context. Additionally, the multi-layer perceptron module was used. The proposed deep model is trained using 22 types of ligneous leaf disease photos from a public dataset. The suggested model’s training time is reduced via transfer learning. Identification of apple leaf diseases is critical for apple production. Propose a new attention strategy to help apple tree growers spot leaf diseases. Cheng and Li present a novel deep learning network based on MobileNet v3 and its methodology. Our network outperformed EfficientNet-B0, ResNet-34, and DenseNet-121 in recognizing apple leaf diseases with a remarkable accuracy of 98.7% on a private dataset. This model also outperforms existing models in accuracy, recall, and f1-score while keeping MobileNet’s fewer parameters and computational efficiency. High-throughput crop monitoring using remotely recorded pictures and deep learning has improved crop health monitoring. Nampally et al. conduct studies on maize crops using various water treatments in a controlled setting. They capture crop data from tillering to heading using a multispectral camera on a UAV. A CNN model was presented with a flexible convolutional layer to learn and extract rich spatial and spectral characteristics. A weighted attention-based bi-directional long short-term memory network processes these features to deal with how they depend on time and order. Aggregated spatial-spectral-temporal Characteristics forecast water stress. To enable more efficient identification of plant diseases and pests, Guan et al. designed a novel network architecture based on EfficientNetV2. The experiments demonstrate that training this model using a dynamic learning rate decay strategy can improve the accuracy of plant disease and pest identification. Transfer learning is incorporated into the training process. After being trained using the dynamic learning rate decay strategy, the model achieves an accuracy of 99.80% on the Plant Village plant disease and pest dataset.

## Image segmentation

Fine ripeness identification can improve strawberry harvest management by providing more precise crop information. Accordingly, Tang et al. offer a technique for recognizing strawberry ripeness in the field. The approach has three steps: after adding self-calibrated convolutions to the Mask R-CNN backbone network to boost model performance, the model extracts the strawberry target from the picture. In the second step, region segmentation divides the strawberry target into four sub-regions and extracts color features. The final step classifies and visualizes strawberry ripeness using color feature values. SVM classifiers provide the best strawberry ripeness classification effect. Classification outperforms manual feature extraction and AlexNet, ResNet18 models. Strawberry improved planting management decisions may be made accurately using this strategy. Precision field segmentation using satellite data is a major difficulty in sugarcane yield prediction and crop management. Yuan et al. propose DSCA-PSPNet using a modified ResNet34 and pyramid scene parsing network with new modules. The proposed sugarcane field feature representation is preferable since it can respond to spatial and channel-wise information.

## Object detection

Jia et al. present an improved YOLOX_m approach for effective green fruit recognition in complicated orchard situations. First, the model uses the CSPDarkNet backbone network to extract three effective feature layers at various sizes from the input picture. These effective feature layers are fed into the feature fusion pyramid network for enhanced feature extraction, which combines feature information from different scales. The Atrous spatial pyramid pooling module increases the receptive field and the network’s ability to obtain multi-scale contextual information. For classification and regression prediction, the head prediction network receives the fused features. Varifocal loss also reduces the influence of an imbalanced positive and negative sample distribution to improve accuracy. Khan et al. explore the use of edge computing devices to improve the accuracy of deep learning models for agricultural applications, while taking into account resource restrictions. Example data came from the publicly accessible Plant Village dataset of healthy and sick leaves for 14 crop species and 6 disease categories. The MobileNetV3-small model achieved 99.50% accuracy in leaf classification. Quantization-based post-training optimization lowered model parameters from 1.5 million to 0.9 million while retaining 99.50% accuracy. The final ONNX model allows deployment on mobile devices and other platforms. It provides a cost-effective way to deploy accurate deep-learning models in agriculture. Vello et al. studied the usefulness of image-based phenotyping using fluorescent and visible light pictures to measure and classify Camelina seeds. They created SeedML, a user-friendly online service that uses phenomics platforms with fluorescent and visible light cameras to detect Camelina seeds from high-salt plants. This gateway can improve quality control, detect stress signs, and track agricultural productivity trends with high throughput. This study may aid climate crisis research and agri-food quality control tool deployment. Mbouembe proposes SBCS-YOLOv5s, an effective tomato identification method. SBCS-YOLOv5s adds SE, BiFPN, CARAFE, and Soft-NMS modules to improve model feature expression. Modelling channel-wise interactions and adaptive re-calibration of feature maps enable the SE attention module to catch essential information and enhance model feature extraction. The SE module’s adaptive re-calibration may also increase model resilience to environmental changes. Next, an efficient, weighted bidirectional feature pyramid network replaced the PANet multi-scale feature fusion network. Third, the neck network replaces the upsampling operator with CARAFE. Better feature maps with more semantic information result from this approach. CARAFE’s spatial detail enhancement helps the model distinguish closely placed fruits. Finally, the Soft-NMS method replaced the Non-Maximum-Suppression (NMS) approach to better identify occluded and overlapping fruits. Soft-NMS’s continuous weighting approach makes it better at managing little and big fruits in images. Yang et al. used a multi-sensor fusion and CNN to identify moisture content in agricultural goods during drying in real time. This work designed a multi-sensor data collection platform and created a CNN prediction model using raw load, air velocity, temperature, tray position data and material weight data. In the model performance comparison, the CNN prediction model had the best prediction effect. Validation trials demonstrated that the detection system satisfied online moisture content detection criteria for agricultural product drying. This work allows online detection of various agricultural product drying indicators. Popescu et al. discuss neural network-based emerging agricultural trends for detecting hazardous insects and pests. Using a systematic review, this technology’s pros and cons and researchers’ methods for improving it are discussed. This review examines pest detection using neural networks, pest databases, current software, and unique modified architectures. Multiple research publications from 2015 to 2022 were analyzed, with fresh patterns analyzed between 2020 and 2022. Molina-Rotger et al. study the use of random forest and support vector machine algorithms to detect and classify olive flies in a Raspberry Pi B+-based electronic trap. Combining the two approaches improves classification accuracy with a limited training data set.

## Monitoring

For ecological fruit production, orchard monitoring is an essential study and practice. Popescu et al. discuss recent advances in orchard monitoring, focusing on neural networks, UAVs, and practical applications. Papers on complicated issues found by combining field keywords were chosen and examined. The study focused on 2017–2022 studies on neural networks and UAVs in orchard monitoring and productivity assessment. UAV trajectories and flights in the orchard were emphasized due to their intricacy. The structure and implementation of the newest neural network systems utilized in such applications, databases, software, and performance are studied. To make recommendations for researchers and end users, the new concepts and their implementations were surveyed in concrete applications.

## Path planning

Zhang et al. offer an enhanced local route planning technique for an artificial potential field, including an elliptic repulsion potential field as the border potential field. The potential field function solves unreachable objectives and local minima by using an enhanced variable polynomial and a distance factor. The scope of the repulsion potential field is changed to an ellipse, and a fruit tree boundary potential field is added, which reduces environmental potential field complexity, allows the robot to avoid obstacles without crossing the fruit tree boundary, and improves its safety when working independently.

All of the 15 accepted papers include advances and novelties in the different topics covered by the Research Topic “*IoT, UAV, BCI empowered deep learning models in precision agriculture*”. The editors are pleased to present this collection of articles to the precision agriculture research area and others related areas, and they hope that it will help researchers’ advances in the future.

## Author contributions

JL: Writing – original draft. JD: Writing – original draft.

